# Vitiligo developing during dupilumab treatment in a child with atopic dermatitis: a case report and literature review

**DOI:** 10.3389/fmed.2026.1947337

**Published:** 2026-09-28

**Authors:** Peng Yu, Fenglei Wei

**Affiliations:** Department of Dermatology, Dalian Women and Children’s Medical Group, Dalian, China

**Keywords:** combination therapy, dupilumab, induce, narrowband-ultraviolet B, vitiligo

## Abstract

An 8-year-11-month-old girl with severe atopic dermatitis had previously received treatments including topical hydrocortisone butyrate, pimecrolimus, tacrolimus, hormone ointment encapsulation, and narrowband ultraviolet B (NB-UVB) phototherapy. She also took oral antihistamines such as cetirizine and loratadine, but these interventions provided limited relief, and her pruritus persisted. In August 2023, she began dupilumab therapy with a 400 mg subcutaneous loading dose, followed by 200 mg subcutaneous injections every two weeks. Vitiligo developed three months after dupilumab initiation in this child with severe atopic dermatitis and improved with combination therapy while dupilumab was continued. Together with previously published reports, this observation supports a possible temporal association between dupilumab exposure and vitiligo development; however, causality cannot be established from the currently available evidence.

## Introduction

Vitiligo, an acquired autoimmune disorder, is distinguished by the gradual destruction of melanocytes in the skin, resulting in patchy loss of pigmentation. Despite its intricate pathogenesis, T lymphocyte-mediated immune responses are pivotal in the progression of vitiligo (1).

This article reports a case of vitiligo-like changes on the hands linked to dupilumab treatment, highlighting the importance for clinicians to be aware of this potential side effect and to use the medication with appropriate caution.

## Case description

An 8-year-11-month-old girl, weighing 35 kg, presented with generalized erythema, scales, papules, and pruritus for over seven years, and leukoderma for three months. She had recurrent episodes of erythematosus papules, papulovesicles, scales, and lichenification, with dry, rough skin and significant pruritus. Despite multiple hospital visits and treatments [topical hydrocortisone butyrate, pimecrolimus, tacrolimus, hormone ointment encapsulation, narrowband ultraviolet B (NB-UVB) phototherapy, and oral antihistamines], these interventions provided limited relief, and the pruritus persisted.

In August 2023, she visited our dermatology clinic with symptoms of dry, rough, itchy skin, accompanied by erythematosus papules, maculopapular rash, nodules, scales, and partial lichenification. The Body Surface Area (BSA) score was 61%, the Scoring Atopic Dermatitis (SCORAD) score was 45.2 points, and the Eczema Area and Severity Index (EASI) score was 15.6 points. Laboratory tests revealed a total IgE level of 684 IU/ml, with positive results for house dust mite (+3) and storage mite (+6). The patient had no history of infection, immune deficiency, or parasitic diseases. There was no personal or family history of vitiligo and no history of drug allergies. However, she had a two-year history of allergic rhinitis and allergic conjunctivitis.

The patient started on dupilumab therapy with a loading dose of 400 mg administered subcutaneously, followed by maintenance injections of 200 mg subcutaneously every two weeks. In November 2023, dermatological examination revealed dry skin with a sandpaper-like texture, residual hyperpigmentation, and vitiligo patches on the dorsum of the second and fourth fingers of the right hand. The vitiligo patches measured the size of a soybean and a fingernail, respectively, with unclear borders and smooth surfaces without scales (Figure 1a). Under Wood's light, porcelain-white fluorescence was observed (Figure 1b). On dermoscopic examination, areas of depigmentation with irregular margins were identified, and hyperpigmentation was noted in the perilesional skin (Figure 1c). The diagnosis was confirmed as atopic dermatitis complicated by vitiligo.

**Figure 1:**
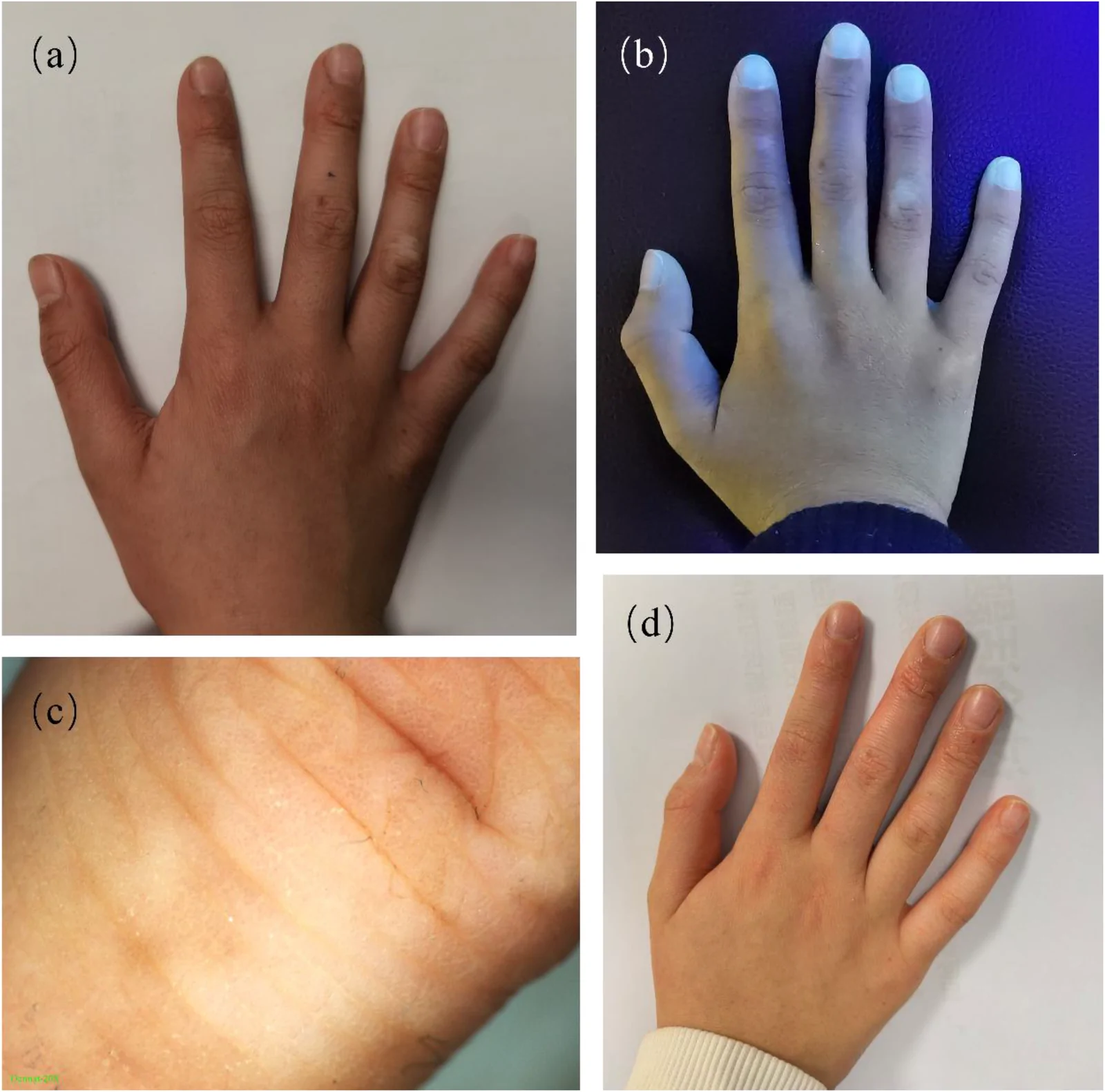
**(a)** right hand, 2nd and 4th fingers: soybean and nail-sized white spots, unclear borders, smooth, no scales. **(b)** Wood's lamp: Porcelain-white fluorescence. **(c)** Dermoscopy (20  ×  ): Irregular depigmented areas, hyperpigmented perilesional skin. **(d)** Vitiligo essentially resolved.

Treatment continued with a dupilumab injection of 200 mg every two weeks subcutaneously. The patient responded favorably to a combination therapy comprising NB-UVB combined with fractional laser therapy and topical corticosteroids. In April 2025, the follow-up visit revealed that the vitiligo had essentially recovered (Figure 1d).

## Discussion

Dupilumab is a fully human monoclonal antibody that selectively targets the IL-4 receptor alpha (IL-4R*α*) subunit, thereby inhibiting the signaling pathways of both interleukin-4 (IL-4) and interleukin-13 (IL-13), and consequently disrupting the downstream inflammatory responses mediated by these cytokines. It has gained approval for the management of moderate to severe atopic dermatitis (2). While common adverse reactions to dupilumab encompass injection site reactions and conjunctivitis, immune-mediated adverse effects, such as psoriasis and alopecia areata, have also been reported (3). The induction of vitiligo-like changes following dupilumab treatment remains a rare but intriguing phenomenon. Recently, several studies have documented cases where dupilumab induced or exacerbated pre-existing vitiligo lesions (4–10).

Our literature search of PubMed, China National Knowledge Infrastructure (CNKI), and Wanfang Data Knowledge Service Platform identified 17 cases from database inception to March 5, 2025. Thirteen patients developed vitiligo-like manifestations after dupilumab treatment, and four experienced worsening of pre-existing vitiligo. Patient characteristics are in Table 1. The management of local skin depigmentation primarily involved topical corticosteroids and phototherapy. Most patients improved within months, regardless of whether they discontinued dupilumab. Depigmentation was more common in exposed skin areas. Vitiligo-like changes or worsening of pre-existing vitiligo usually occurred within six months of starting dupilumab treatment.

**Table 1:** Published reports of new-onset vitiligo-like changes or worsening of pre-existing vitiligo during dupilumab treatment.

Reports	Gender	Age (years)	Dupilumab indication	Previous history of vitiligo	Duration from dupilumab initiation to vitiligo onset (months)	Distribution of vitiligo	Dupilumab cessation	Treatment	Vitiligo outcome	Remission duration of vitiligo (months)
Ferreirinha et al., 2024 (7)	Female	30	AD	No	24	Armpits and elbows	No	Tacrolimus 0.1% ointment plus NB-UVB	Better	6
	Male	20	AD	No	3	Wrists and ankles	Yes	Tacrolimus 0.1% ointment	Stable	3
Shao et al., 2024 (9)	Male	80	AD	No	2	Chest, back, and lower limbs	Yes	Glycyrrhizin	Stable	Unreported
	Female	14	AD	No	1	Forehead	No	Hydrocortisone butyrate ointment	Better	5
Bian et al., 2024 (10)	Female	10	AD	No	10	Upper extremities	Yes	Tacrolimus 0.1% ointment and topical corticosteroids	Basic recovery	4
Picone et al., 2023 (8)	Male	79	AD	No	1	Scalp, neck, and back of the hands	No	Topical corticosteroids and NB-UVB	Better	4
Ren et al., 2023 (5)	Male	52	NP	Yes	3	Forehead, glabella, nose, and cheeks	No	Ruxolitinib 1.5% cream and NB-UVB	Stable	Unreported
	Female	43	NP	Yes	1	Face, bilateral breasts, chest, abdomen, bilateral forearms, and proximal thighs	No	Betamethasone dipropionate 0.05% cream, tacrolimus 0.1% ointment, and NB-UVB	Stable	Unreported
	Female	18	AD	Yes	4	Pelvis, left leg, and lower torso	No	Triamcinolone acetonide 0.1% ointment	Resolution	Unreported
	Male	50	AD	No	3	Right hairline, neck, and chest	No	Triamcinolone acetonide 0.1% ointment and tacrolimus 0.1%	Resolution	Unreported
	Female	40	AD	No	5	Hands and feet	No	None	Resolution	Unreported
	Male	18	AD	No	2	Bilateral cheeks, neck	Yes	Tacrolimus 0.1% ointment	Ongoing	Unreported
	Female	50	AD	No	6	Right hairline, neck, and chest	Yes	Mometasone 0.1% ointment and NB-UVB	Resolution	Unreported
Napolitano et al., 2023 (3)	Male Female	^a^52 ± 38.18	AD	No	1.97 ± 2.67	Unreported	No	Topical corticosteroids and NB-UVB	Better	4.31 ± 2.67
Huang et al., 2022 (6)	Male	72	AD	No	1.75	Right forehead	No	None	Stable	Unreported
Takeoka et al., 2021 (4)	Male	17	AD	Yes	3	Forehead, partial hair	Yes	Topical corticosteroids and NB-UVB	Better	17

AD, atopic dermatitis; NP, nasal polyps; NB-UVB, narrowband-ultraviolet B; ^a^The literature reported the average age of the two patients but did not specify the individual ages.

We reported the youngest patient with dupilumab-induced vitiligo-like changes, presenting as non-segmental lesions on the dorsal hand. Vitiligo appeared three months after dupilumab injection, consistent with prior studies (4–9). The patient responded well to combination therapy with NB-UVB, fractional laser, and topical corticosteroids, achieving pigment restoration after 16 months.

## Comparison with previous reports and interpretation of the possible association

The present case should be interpreted in the context of the limited and heterogeneous literature on vitiligo developing during dupilumab treatment. Previous reports include both new-onset vitiligo-like lesions and exacerbation of pre-existing vitiligo, indicating that the clinical presentation may differ according to individual susceptibility and baseline immune status (4, 5, 7, 8, 10). Most reported patients were treated with dupilumab for atopic dermatitis, although the available evidence is based predominantly on isolated case reports and small case series. Therefore, the current literature should be interpreted as a potential pharmacovigilance signal rather than definitive evidence of a causal adverse drug reaction.

In the present case, depigmented lesions developed three months after dupilumab initiation. This timing is consistent with previous reports, in which vitiligo or vitiligo-like changes generally appeared during the first six months of treatment. To our knowledge, our patient, aged 8 years and 11 months, is the youngest reported patient with vitiligo developing during dupilumab treatment. This observation extends the existing evidence to a younger pediatric population. However, a temporal relationship alone cannot establish that dupilumab directly caused the vitiligo.

The localized distribution of lesions on the dorsal fingers is consistent with reports describing involvement of exposed sites, including the hands, face, scalp, and neck, but differs from cases with extensive truncal or limb involvement. This heterogeneity may reflect variation in individual autoimmune susceptibility, pre-existing subclinical melanocyte-directed immunity, atopic dermatitis severity, environmental triggers, or concomitant treatments. These factors could not be assessed fully in the present case and remain important potential confounders. The outcome in our patient also requires cautious interpretation. Pigment restoration was observed while dupilumab was continued, together with narrowband ultraviolet B phototherapy, fractional laser therapy, and topical corticosteroids. This course is consistent with some previous reports in which vitiligo improved or remained stable despite continued dupilumab treatment. In contrast, dupilumab was discontinued in other reported cases. Because treatment strategies and outcomes differ substantially among cases, it is not possible to determine whether improvement resulted from dupilumab continuation or discontinuation, vitiligo-directed treatment, spontaneous disease stabilization, or a combination of these factors.

A possible mechanistic explanation is that IL-4/IL-13 blockade may modify the balance between type 2 and Th1/Th17-related immune pathways. Since vitiligo is associated with IFN-*γ* signaling, autoreactive CD8+ T cells, and melanocyte destruction, such an immune shift could hypothetically contribute to the onset or unmasking of vitiligo in predisposed individuals. Nevertheless, this explanation remains speculative. Cytokine assessment, melanocyte-specific autoantibody testing, genetic susceptibility analysis, and lesional immunohistochemistry were not performed in the present patient. Further controlled observational studies and mechanistic investigations are required to determine whether dupilumab increases vitiligo risk and to identify patients who may be susceptible.

Emerging pharmacovigilance evidence provides additional support for a potential association between type 2 immune-response inhibitors and reported vitiligo. A recent analysis of the FDA Adverse Event Reporting System identified 86 vitiligo reports associated with type 2 immune-response inhibitors between January 2004 and September 2024. Dupilumab was the most frequently reported agent, accounting for 81.4% of reports, and showed a positive disproportionality signal for vitiligo reporting (reporting odds ratio 2.67, 95% confidence interval 2.11–3.40). A broader FAERS pharmacovigilance analysis also identified dupilumab among drugs with a positive vitiligo-reporting signal. However, spontaneous-reporting data cannot establish incidence or causality because FAERS is susceptible to reporting bias, incomplete clinical information, duplicate reports, and the absence of a reliable exposure denominator. These findings should therefore be interpreted as a pharmacovigilance signal that warrants further investigation rather than proof of a causal adverse effect (11, 12).

An important alternative explanation is the background autoimmune susceptibility associated with atopic dermatitis itself. Patients receiving dupilumab are not randomly selected from the general population; they usually have moderate-to-severe atopic dermatitis and may already have an increased baseline risk of autoimmune diseases, including vitiligo. A systematic review and meta-analysis reported a significant association between atopic dermatitis and vitiligo, with an odds ratio of 3.21 in case-control and cross-sectional studies and a relative risk of 1.64 in cohort studies. Other meta-analyses have similarly reported an increased risk of autoimmune diseases among patients with atopic dermatitis. Therefore, vitiligo occurring after dupilumab initiation may represent spontaneous disease development within an autoimmune-prone population, a drug-facilitated event, or a combination of both. This distinction cannot be resolved by case reports and requires controlled studies comparing patients with atopic dermatitis of similar severity who are exposed and unexposed to dupilumab (13–15).

Vitiligo is characterized by localized depigmented patches due to the absence of functional melanocytes, with a complex pathogenesis involving Th1-mediated immune responses (7). IL-4 limits the conversion of naive T cells to Th1 (16, 17), inhibiting IFN-*γ* production and suppressing CD8+ T cell responses (18). During dupilumab treatment, IL-4 inhibition may expand Th1 and Th17 pathways, increasing cytokines like IFN-*γ*, IL-17, IL-2, and TNF-α, potentially contributing to vitiligo (7, 19).

IFN-*γ* is a key Th1 cytokine that plays a crucial role in initiating vitiligo. Its elevated expression in lesional skin and plasma suggests it could be a valuable biomarker for disease activity and prognosis. Studies show that TNF-α levels correlate with disease activity in localized vitiligo, while IL-17 levels correlate with affected body surface area in generalized vitiligo (20) Autoreactive CD8+ T cells, recruited via the Th1/Th17 pathway, destroy melanocytes. IFN-*γ* promotes the recruitment and accumulation of these CD8+ T cells in the skin, leading to melanocyte apoptosis (21).

This report has several limitations. First, a single case report cannot establish incidence, relative risk, or direct causality. Second, the currently available literature consists mainly of isolated case reports and small case series, which are vulnerable to reporting and publication bias and do not include disease-matched untreated control groups. Third, atopic dermatitis itself is associated with immune dysregulation, and the contribution of underlying autoimmune susceptibility, subclinical pre-existing vitiligo, genetic predisposition, environmental triggers, or previous treatments cannot be excluded. Fourth, our patient received narrowband ultraviolet B phototherapy, fractional laser therapy, and topical corticosteroids after vitiligo onset; therefore, the relative contribution of each treatment to repigmentation cannot be determined. Finally, cytokine profiling, immunological testing, genetic analysis, melanocyte-specific autoantibody assessment, and lesional immunohistochemistry were not performed. Accordingly, this case demonstrates a temporal association between dupilumab exposure and vitiligo development but cannot confirm a direct causal relationship.

This case expands the limited literature on vitiligo developing during dupilumab treatment and, to our knowledge, describes the youngest reported patient to date. The temporal relationship, together with similar published observations and the biological plausibility of immune-pathway modulation, supports continued clinical awareness and pharmacovigilance. However, this report does not establish that dupilumab directly caused vitiligo. Underlying atopic dermatitis-related immune dysregulation, individual autoimmune or genetic susceptibility, environmental triggers, and concomitant treatments may have contributed. Prospective controlled studies with detailed clinical, immunological, and genetic characterization are needed to determine whether dupilumab increases vitiligo risk and to identify patients who may be susceptible.

## Data Availability

The raw data supporting the conclusions of this article will be made available by the authors, without undue reservation.
